# Effects of power training in older patients with multiple sclerosis on neurodegeneration, neuromuscular function, and physical function. A study protocol for the “power training in older multiple sclerosis patients (PoTOMS) randomized control trial

**DOI:** 10.1016/j.conctc.2024.101279

**Published:** 2024-02-19

**Authors:** Tobias Gaemelke, Christoffer Laustsen, Peter Feys, Lars Folkestad, Marianne Skovsager Andersen, Niklas Rye Jørgensen, Marie-Louise Jørgensen, Sune Nørhøj Jespersen, Steffen Ringgaard, Simon F. Eskildsen, Ulrik Dalgas, Lars G. Hvid

**Affiliations:** a Exercise Biology, Department of Public Health, Aarhus University, Aarhus, Denmark; b The MR Research Center, Department of Clinical Medicine, Aarhus University, Aarhus, Denmark; c REVAL, Rehabilitation Research Center, BIOMED, Biomedical Research Institute, Faculty of Medicine and Life Sciences, Hasselt University, Hasselt, Belgium; d Department of Endocrinology, Odense University Hospital and University of Southern Denmark, Odense, Denmark; e Department of Clinical Biochemistry, Rigshospitalet, Copenhagen, Denmark; f Department of Clinical Medicine, Faculty of Health and Medical Sciences, University of Copenhagen, Copenhagen, Denmark; g School of Applied Sciences, Business Academy Aarhus, Aarhus, Denmark; h Department of Physics and Astronomy, Aarhus University, Aarhus, Denmark; i Center of Functionally Integrative Neuroscience and MINDLab, Department of Clinical Medicine, Aarhus University, Aarhus, Denmark; j The Danish MS Hospitals, Ry and Haslev, Denmark

**Keywords:** Multiple sclerosis, resistance exercise, aging, neuroprotection

## Abstract

**Introduction:**

Approximately one-third of all persons with multiple sclerosis (pwMS) are older, i.e., having an age ≥60 years. Whilst ageing and MS separately elicit deteriorating effects on brain morphology, neuromuscular function, and physical function, the combination of ageing and MS may pose a particular challenge. To counteract such detrimental changes, power training (i.e., a type of resistance exercise focusing on moderate-to-high loading at maximal intended movement velocity) presents itself as a viable and highly effective solution. Power training is known to positively impact physical function, neuromuscular function, as well as brain morphology. Existing evidence is promising but limited to young and middle-aged pwMS, with the effects of power training remaining to be elucidated in older pwMS.

**Methods:**

The presented ‘Power Training in Older MS patients (PoTOMS)’ trial is a national, multi-center, parallel-group, randomized controlled trial. The trial compares 24 weeks of usual care(n = 30) to 24 weeks of usual care and power training (n = 30). The primary outcome is whole brain atrophy rate. The secondary outcomes include changes in brain micro and macro structures, neuromuscular function, physical function, cognitive function, bone health, and patient-reported outcomes.

**Ethics and dissemination:**

The presented study is approved by The Regional Ethics Committee (reference number 1-10-72-222-20) and registered at the Danish Data Protection Agency (reference number 2016-051-000001). All study findings will be published in scientific peer-reviewed journals and presented at relevant scientific conferences independent of the results. The www.clinicaltrials.gov identifier is NCT04762342.

## Highlights

• The ‘Power Training in Older Multiple Sclerosis patients (PoTOMS)’ trial investigate the effect of 24 weeks power training on neuroimaging in older people with multiple sclerosis.
• Extensive testing will provide a comprehensive understanding of the potential effects of power training in this population.
• A 24-week follow-up period allows for an investigation of the sustainability of potential effects of power training.

## 1 Introduction

Multiple sclerosis (MS) is characterized by focal lesions and whole brain atrophy caused by an autoimmune dysfunction [1]. Such neurodegeneration, quantifiable by MRI [2], results in a plethora of symptoms including motor, cognitive, and sensory impairments [1], affecting the lives of people with MS (pwMS) [3]. Importantly, the prevalence of older (≥60 years of age) pwMS has increased substantially and will expectedly continue to do so in the coming decades [[4], [5], [6], [7]]. Today, approximately 1/3 of all pwMS are older [[7], [8], [9]]. This introduces a combined challenge comprised of ageing and MS, which separately have deteriorating effects on whole brain volume [[10], [11], [12], [13], [14]], regional brain volume [[12], [13], [14], [15], [16]], physical function [[17], [18], [19], [20]], neuromuscular function [[21], [22], [23], [24]], and bone health [25,26].

Although sparsely investigated, the combined effect of MS and ageing is known to impair walking capacity [[27], [28], [29], [30], [31]], lower limb neuromuscular function [30] including muscle power [29], and increase frailty [32,33] in older pwMS when compared to age-matched healthy controls (HC). This limited physical function is paralleled by the reduced self-reported walking ability of older pwMS [27,34,35]. Unfortunately, there is a paucity of research related to potential interventions counteracting the combined deleterious consequences of ageing and MS, making further research into this MS subgroup highly warranted.

Progressive resistance exercise (PRE) has resulted in improved physical and neuromuscular function in young and middle-aged pwMS [36,37], with dynamic muscle strength as well as rate of force development (RFD) being particularly susceptible towards adaptation [21]. Of note, such adaptations are predominantly attributed to functional adaptations of the CNS, with improved neural drive [[38], [39], [40]]. In addition, data from a pilot randomized controlled crossover study involving n = 35 pwMS suggests that 24 weeks of PRE improves cortical thickness and reported a trend towards reduced whole brain atrophy [41]. Despite these positive effects of PRE in pwMS, it has not yet been investigated in older pwMS; however, data from older healthy individuals show that PRE induces positive effects on both physical function [42,43] and neuromuscular function [42,44]. This is mainly achieved through increased neural drive to the muscles [42,45] as well as muscle hypertrophy [46,47]. The former is suggestive of an improved CNS ability to recruit and fire motor neurons following PRE.

PRE can be performed as progressive power training (PPT; PRE performed with moderate-to-high loading at maximal concentric movement velocity) hereby increasing demands of the CNS to activate the muscle. PPT induces comparable improvements in muscle strength as traditional PRE, but greater improvements in muscle power [48,49]. This is important, as pwMS have substantial muscle strength reductions during fast concentric contractions [21] and since muscle power, more so than muscle force, is a strong predictor of physical function in older adults [50,51]; nevertheless, power training has only been sparsely investigated in pwMS so far [52], and still not in older pwMS where it may have the most profound effects.

Consequently, the primary aim of the present study is to compare the effects of 24 weeks of power training to a “usual care” control group on neurodegeneration (i.e., annualized whole brain atrophy rate) in older pwMS (≥60 years). A secondary aim is to evaluate the effects of 24 weeks power training on additional macro and micro brain structures, neuromuscular function, physical function, cognitive function, bone health and patient-reported outcomes.

For the primary aim, we test the hypothesis that older pwMS performing 24 weeks of power training will elicit a reduced annualized whole brain atrophy rate compared with the control group. For the secondary aim we test the hypothesis that performing 24 weeks of power training will improve brain micro and macro structures, neuromuscular, physical, cognitive function, bone health, and patient-reported outcomes.

## 2 Methods

### 2.1 Study design

**Fig. 1:**
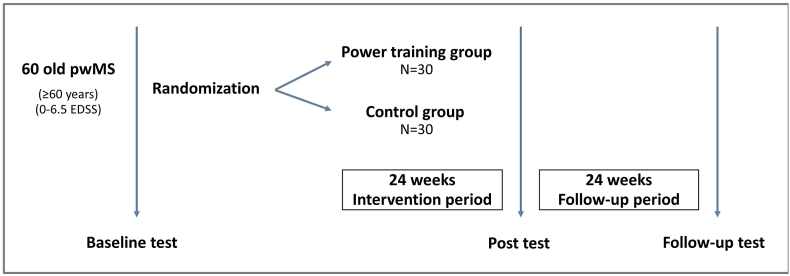
Study design of the PoTOMS trial.

The PoTOMS trial is a 24-week national, multi-center, parallel group, randomized controlled trial (RCT) with 24-weeks of follow-up. The present study compares an intervention group of older pwMS receiving 24 weeks of high-load progressive power training (PPT) to a control group of older pwMS receiving usual care, comprising standard healthcare provided to pwMS within the Danish healthcare system. The study design of the PoTOMS trial is depicted in Fig. 1. This study protocol conforms to the SPIRIT statement, and the trial is registered at www.clinicaltrials.gov (registration number NCT04762342).

### 2.2 Recruitment and eligibility

PwMS will be recruited via four Danish regional MS clinics (Aarhus University Hospital, Esbjerg Hospital, Odense University Hospital, and Rigshospitalet), events hosted by the Danish MS Society, www.forsøgsperson.dk, and social media. All pwMS will be supplied with a leaflet explaining the study (design and content) and an invitation to participate. Interested pwMS will then receive detailed written study information and a leaflet from the national ethical committee explaining the rights of participants in health science research projects according to Danish legislation. The project coordinator will screen potential participants according to the following inclusion and exclusion criteria. Older pwMS will be eligible for inclusion if they are:

- ≥ 60 years of age.
- clinically diagnosed with MS according to the McDonald criteria [53].
- having an EDSS ≤6.5.
- capable of self-transportation to testing at Aarhus University and Aarhus University Hospital.
- capable of self-transportation to training, if randomized to the PPT group.

PwMS will be excluded if they are:

- having comorbidities (cardiovascular, respiratory, orthopedic, or other neurological diseases than MS) affecting PPT participation or testing.
- having a pacemaker.
- having metallic implant(s) that prevents MRI scans.
- having untreated osteoporosis; t-score below −2.5 and a history of low energy fracture or t-score below −3.0.
- participating in ≥2 sessions per week of structured PRE and have done so for the past 3 months.
- having cognitive impairments that prevent the participant from understanding training and testing instructions.

All participants will provide informed consent to the trial leader (TG) before participation.

### 2.3 Sample size

The present study is powered based on the primary outcome; percentage total brain volume change after 24 weeks of PRE in pwMS quantified by structural image evaluation, using normalization, of atrophy (SIENA) [41] and the assumption that PPT is ∼15% more effective than traditional of PRE [49,54]. A two-sample two-sided power calculation showed that a total of 30 subjects are needed in each group to adequately power the study (α = 0.05; power = 0.8; control mean = 0.3204 (SD 0.5253); exercise mean = −0.0148 (SD 0.2698); dropout rate = 16%).

### 2.4 Random allocation procedure

Randomisation to either the PPT group or the control group will be performed after baseline testing by the project coordinator in a 1:1 allocation ratio. Allocation is done by the sealed envelope principle.

### 2.5 Intervention

#### 2.5.1 High-load progressive power training

PwMS randomized to the PPT group will receive 24 weeks of supervised PPT with two weekly sessions and usual care. To improve adherence to PPT, four sites delivering exercise will be established in the cities where the recruiting MS centres are located (Aarhus, Copenhagen, Esbjerg, and Odense). The PPT intervention will be delivered at university fitness facilities and supervised by exercise physiology students who, besides their educational background, will be thoroughly educated and instructed in how to deliver the specific PPT program. All trainers will receive a detailed booklet describing the training program and related procedures; further, a ‘hotline’ to the lead researcher exists if any questions should arise. Education and instruction of the trainers include correct performance of exercises and safety measures regarding training older pwMS [55,56]. Training sessions will throughout the intervention period, be offered 5 days a week. The intention is to carry out all sessions in small groups without surpassing a 4:1 ratio of participants to the supervising trainer. Individual sessions will nevertheless be carried out if required.

An overview of the PPT intervention can be seen in Table 1. The first four weeks of the PPT program serve as an introduction, focusing on the correct execution of exercises. Weeks 5–8 emphasise the performance of exercises with the correct muscle contraction velocity, which is essential for PPT, and this exercise manipulation is used in the remaining weeks. During weeks 9–24 additional, translational power exercises also involving power training aspects are added to the program (referred to as ‘translational mesocycle blocks’). This small part of the PPT program is aimed at facilitating the translation of exercise adaptions to daily physical function. The translational mesocycle blocks consist of exercises that include walking accelerations, fast chair raises, and balancing exercises. There is also some upper body PPT added. The translational mesocycle is separated into 4 blocks lasting 4 weeks each. Blocks 1 and 3 and 2 and 4, respectively, share the same exercises, except that the relative intensity and volume are progressed from block 1 to 3 and from block 2 to 4. Altogether The PPT program targets the whole body, yet with a primary focus on the lower extremities.

**Table 1:**
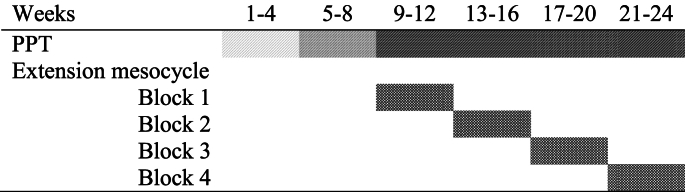
Overview of the PPT intervention.

Introduction to resistance exercise (light grey marking), introduction to PPT (grey marking), PPT (dark grey marking), and extension mesocycle (crossed lines). The extension mesocycle blocks adds exercises such as intermittent walking to the intervention. Abbreviations: PPT: High-load progressive power training.

*Warm-up:* Each session begins with a warm-up, consisting of ergometer cycling for 2 min at low intensity directly followed by 2 min at moderate intensity. Subsequently, standing balance is performed in 2 sets of 15 s for each leg. The balance exercises are gradually progressed by reducing the level of support when possible (e.g., advancing from supporting with two hands to one hand). To finalize the warm-up, participants perform 2 × 30 s alternating knee raises where the anterior thigh reaches horizontal. When participants can perform ≥26 repetitions, ankle weights are added with 1 kg increments for each leg.

*PPT:* After the warm-up, the PPT follows (see description in Table 2), which consists of five exercises performed in machines (bilateral leg-press, bilateral plantar flexion performed in leg-press machine), unilateral knee extension, bilateral lying leg curl) and one exercise performed with an elastic band (unilateral banded dorsal flexion). All exercises, both bilateral and unilateral, are performed with the intent to match the relative intensity in both limbs. The PPT program is planned so that the exercises are performed in three mesocycles: First as 3 sets of 12 repetitions at 14-RM during weeks 1–8; second as 3 sets of 10 repetitions at 12-RM during weeks 9–16; and third as 3 sets of 8 repetitions at 10-RM during weeks 17–24. Progression of external load is performed when a participant can perform ≥2 repetitions more than planned according to participants own rate of perceived exertion (RPE) scale, ranging from 0 to 10 (10 being maximal exertion) [57]. Unilateral banded dorsal flexion is based on band tension as external resistance and hence load is solely determined by RPE. Here participants are expected to reach an RPE of 7-to-9.5.

**Table 2:** Detailed description of the PPT intervention.

Load (RM)	14 (week 1–8), 12 (week 9–16), 10 (week 17–24)	
Repetitions (number)	12 (week 1–8), 10 (week 9–16), 8 (week 17–24)	
Sets (number)	3	
Rest between sets (min)	1½	
Session duration (min)	60	
Duration of training period (weeks)	24	
Time between sessions (hours)	≥48	
Exercise manipulation	Traditional PRE (week 1–4), PPT (week 5–24)	
Velocity of PTT	The concentric contraction is performed as fast as possible, and the eccentric contraction duration is 1–2s	
Exercise	Loading	Performed in week
Bilateral leg-press	Weight stack machine	1–24
Bilateral plantar flexion	Weight stack machine	1–24
Bilateral knee extension	Weight stack machine	1–24
Unilateral banded dorsal flexion	Elastic band	1–24
Bilateral lying leg curl	Weight stack machine	1–24
Back extension	Weight vest	1–24
Shoulder press	Dumbbell	9-12, 17-20
Seated row	Weight stack machine	9-12, 17-20
Chest press	Weight stack machine	13-16, 21-24
Lat pull-down	Weight stack machine	13-16, 21-24

Abbreviations: PPT: High-load progressive power training. RM: Repetition maximum, PRE: Progressive resistance exercise.

Evaluation of progression in training exercises is done by a 5 R M test performed in bilateral leg-press and unilateral knee extension in week 1 (session 2), week 5 (session 9), week 9 (session 17), week 13 (session 25), week 17 (session 33), and week 21 (session 41).

*Translational extension mesocycle:* The exercises added in the four blocks of ‘translational extension mesocycle’ are walking drills, dumbbell shoulder press, seated row, chair rise, countermovement jump, chest press, lat. pull-down, and interval walking. These exercises are added to facilitate the translation of PPT effects to daily activities and to secure variation of the training to facilitate adherence.

Documentation of PPT is performed by registering the number of performed training sessions, repetitions, sets, external load, and RPE. Session adherence to PPT is evaluated as number of sessions attended, expressed as a percentage of the total number of planned sessions [58]. Content adherence to the intervention is measured as number of repetitions performed in the PPT expressed as a percentage of the total number of planned repetitions. Per protocol completion of the exercise intervention is defined as 40 or more of the 48 planned sessions and an exercise session is considered completed with an ≥80% content adherence.

#### 2.5.2 Control group

To evaluate effects of the PPT intervention in older pwMS, we will compare it with a group of older pwMS receiving usual care only, thus serving as the control group. Usual care encompasses the health care services offered for pwMS within the Danish health care system. The specific health care offered depends on various factors, including disability status, and disease severity, but could include services as disease-modifying treatments, symptom management, various aids (ranging from walking aids to caretakers), and rehabilitation. The control group will not receive any additional intervention but are allowed to continue their usual activities (or start new activities if desired).

### 2.6 Outcomes

All outcomes will be assessed at week 0, 24, and 48. An overview of outcomes can be seen in Table 3.

**Table 3:** Overview of test.

	Pre intervention test	Post intervention test	Follow-up test
Brain MRI			
MP2RAGE	x	x	x
T2 Space, dark fluid	x	x	x
DKI	x	x	x
Leg MRI			
Leg 2D Dixon	x	x	x
Muscle strength			
Bilateral leg press	x	x	x
Hand grip	x	x	x
Knee extension	x	x	x
Knee flexion	x	x	x
Plantar ankle flexion	x	x	x
Dorsal ankle flexion	x	x	x
Neural function			
ITT	x	x	x
EMG	x	x	x
Cognition			
SRT	x	x	x
SDMT	x	x	x
Clinical disability			
EDSS	x		
Physical function			
T25FWT	x	x	x
SSST	x	x	x
6MWT	x	x	x
9SSA	x	x	x
NHPT	x	x	x
1STS	x	x	x
5STS	x	x	x
Standing balance	x	x	x
Blood sample			
Neurodegenerative factors	x	x	x
Neurotrophic factors	x	x	x
Immunological factors	x	x	x
Bone metabolism	x	x	x
Body composition			
DXA	x	x	x
Physical activity			
Accelerometry	x	x	x
Questionnaires			
MSIS	x	x	x
MSWS	x	x	x
HADS	x	x	x
MFIS	x	x	x
EQ5D	x	x	x
FES-I	x	x	x
PSQI	x	x	x
ADL	x	x	x
BPI	x	x	x

Abbreviations: MP2RAGE: Magnetization Prepared 2 Rapid Acquisition Gradient Echo, DKI: Diffusion kurtosis imaging, T25FWT: Timed 25-foot walk test, SSST: Six spot step test, 6MWT: Six-minute walk test, 9SSA: Nine step stair accent, NHPT: Nine-hole peg test, 1STS: one time sit-to-stand test, 5STS: five time sit-to-stand test, SRT: Selective reminding test, SDMT: Symbol digit modality test, EDSS: Expanded disability status scale, DXA: Dual-energy X-ray absorptiometry MSIS: Multiple sclerosis impact scale, MSWS: Multiple sclerosis walking scale, MFIS: Modified fatigue impact scale, HADS: Hospital anxiety and depression scale, EQ-5D: Euro quality of life 5-dimension, FES-I: Falls efficacy scale-international, PSQI: Pittsburgh sleep quality index, ADL: Activities of daily living, BPI: Brief pain inventory.

#### 2.6.1 Primary outcome

The primary outcome of the study is MRI-derived whole brain atrophy rate measured using SIENA [59]. All MRI scans will be performed on the same 3-T MRI scanner (MAGNETOM Skyra, Siemens Medical Systems, Erlangen, Germany) at baseline, 24 weeks (post-intervention), and 48 weeks (follow-up). MRI analyses will be performed by a blinded assessor. Annualized whole brain atrophy rate is quantified using structural T1-weighted 3D Magnetization Prepared 2 Rapid Acquisition Gradient Echo images [60] acquired with a Field-of-view (FOV) of 256 × 256 mm^2^, matrix of 256 × 256, spatial resolution of 1 × 1 × 1 mm^3^. Repetition time (TR) is 5000 ms, Echo time (TE) is 2.98 ms and Inversion time (TI) is 707 ms/2500 ms. The image processing will follow the steps outlined by Riemenschneider et al. [61].

#### 2.6.2 Secondary outcomes

Analysis of macrostructural changes of brain tissue based on T1 weighted images consists of percentage change in brain parenchymal fraction, white matter volume, grey matter volume, and cortical thickness using FACE [62]. Whole brain white matter lesion load and lesion count are derived from fluid attenuated inversion recovery (FLAIR) images using LST [63]. The FLAIR sequence is a 3D SPACE sequence with FOV = 250 × 364 mm^2^, matrix = 220 × 320, and spatial resolution = 1.1 × 1.1 × 1.2 mm^3^. TR/TE/TI = 5000/386/1800 ms.

To study brain microstructure a diffusion kurtosis imaging (DKI) sequence is applied. From this, a range of parameters describing the tissue microstructure can be derived. These include fractional anisotropy, mean diffusivity, axial diffusivity, radial diffusivity, axonal signal fraction, intra-axonal diffusivity, extra-axonal axial diffusivity, and extra-axonal radial diffusivity. Finally, the DKI sequence is utilized to measure white matter fibre orientations by calculating tractography maps. These are important for understanding the association between functional symptoms and structural connections affected by white matter lesions. DKI is based on single-shot diffusion weighted EPI sequence with b-values of 250, 1000 and 2000 s/mm^2^ and 84 diffusion directions in total. FOV is 216 × 216 mm^2^, matrix is 128 × 128, and spatial resolution is 1.7 × 1.7 mm^2^ with a slice thickness of 3 mm. TR/TE is 9300/97 ms.

The PPT intervention is expected to affect brain regions involved in motor function [[64], [65], [66], [67], [68], [69], [70]]. Thus, a separate analysis for these regions is conducted. The following nuclei are segmented and processed to study changes in macro- and microstructure: thalamus, hippocampus, putamen, caudate, globus pallidus, corticospinal tract, cingulate gyrus, corpus callosum, cervical spinal volume, and motor cortex.

A multi-slice 2D, T1-weighted spin echo DIXON sequence is acquired bilaterally for the leg and thigh. Thirty slices with a thickness of 3 mm will be acquired on the thigh and the calf separately. FOV is 450 × 309 mm^2^, matrix is 640 × 352, and spatial resolution is 0.70 × 0.88 mm^2^. TR/TE is 1260/11 ms. Using ITK snap (version 3.8.0 or higher) regions of interest are drawn using the high resolution and contrast DIXON sequence. The region of interest includes the rectus femoris, vastus medialis, vastus lateralis, vastus intermedius, gastrocnemius, soleus, and tibialis anterior. From these segmentations, analysis of muscle cross-sectional area is performed in ITK snap.

##### 2.6.2.1 Physical functional

The timed 25-foot walk test (T25FWT), is a validated measure of maximal walking speed in pwMS [71]. It is performed on a clearly marked 25-foot course and participants are instructed to walk 25 feet as walk twice at their usual walking speed and twice as fast (although safe) as possible [71]. The result is the average time of the two walking tasks. A change exceeding 20% is considered a clinically meaningful change for the T25FWT [72].

The 6-minute walk test (6MWT) is a measure of walking endurance in pwMS [73,74]. The test is performed in a 30 m hallway [73] in accordance with the ATS statement script [75] and participants are instructed to walk as fast (although safe) as possible. A clinically meaningful change in distance walked in the 6MWT is 21.6 m or more in pwMS [74]. Additionally, the 6MWT also determines motor fatigability in pwMS following the procedures previously described by Leone et al. [76].

The six-sport step test (SSST) measures complex walking [77] and requires changes in direction, balance, and coordination. The SSST have sufficient construct validity and discriminative properties [78], test–retest agreement, and reliability [79]. The test is performed in accordance with the original script of Nieuwenhius et al. [77] applying the adjustments proposed by Callesen et al. [79]. The test is conducted on a 1 × 5 m rectangular course where the patient walked as fast (although safe) as possible while pushing five wooden blocks out of circles marked on the floor. The test is performed two times with each foot, and the score is the average time of the four tries [77].

The 9-step stair accent (9SSA) test reflects an important task of daily living. The test is performed using an indoor standard flight of stairs (depth of 29 cm and height of 17 cm). Participants are instructed to ascend the 9 steps one step at a time as fast (although safe) as possible [30]. The use of railing and/or their assistive device is allowed if needed. Two trials of stair climbing are performed, using the best trial as the result.

The 5-times sit-to-stand (5STS) and 1-time sit-to-stand (1STS) measures chair rise capacity [80] and functional lower extremity muscle power [29]. Participants are instructed to rise from a seated position and sit back down five times as fast as possible, yet safely. Then, participants are instructed to perform one maximal chair rise. Both chair rise tests are performed according to the script of Møller et al. [80]. A linear encoder (CRONOJUMP, Bosco system, v1.8.1, Spain; sampling rate 1000 Hz) is fastened to a belt placed on the hip of the participant and provides displacement of the approximated centre of mass of the participant during the 1STS. The latter follows the procedure described by Stagsted et al. [29]. The participants complete two trials and the best serve as the final test result and results are reported for 5STS as time to completion and average power and peak power for 1STS.

The nine-hole peg test (9HPT) measures manual dexterity and upper extremity function. It is conducted with the 9HPT testing device consisting of a container, nine pegs, and nine holes in a symmetrical square pattern. The participants are instructed to insert the nine pegs one by one into each of the nine holes and then remove them again individually, as fast as possible. The test is performed twice with both the dominant and non-dominant hand. The average time of all four trials is used as the outcome [81]. A clinically meaningful change in the 9HPT is an improvement of 20% or more [82].

Standing balance is measured in accordance with the script of the short physical performance battery [83]. Here participants are asked to balance with their feet together side-by-side for 10 s and if successful advance to standing in semi-tandem, in tandem, and using only the weakest leg. If the participants fail to keep 10 s of balance in one stance the test will not proceed to subsequent stands.

##### 2.6.2.2 Neuromuscular function

Test of muscle strength is performed using three dynamometers: A custom build digital leg-press dynamometer, a custom build digital handgrip dynamometer, and an isokinetic dynamometer (Humac Norm, CSMi, Stoughton, MA). Prior to testing, all dynamometers will be calibrated. During all trials, participants are instructed to contract as hard and fast as possible whilst receiving verbal encouragement along with visual feedback to motivate participants to perform at maximal intensity.

In the leg-press dynamometer, isometric MVC is assessed unilaterally in a seated position with 15° knee flexion and 100° hip flexion. Participants are instructed to sit with their arms crossed over their chest. Three trials are performed with each leg or until no increase in maximum force is produced and the trial with the highest MVC is chosen for further analysis. In addition to leg-press MVC, force steadiness is also assessed during leg-press, which is a measure known to be related to motor function (e.g., walking) [84,85]. To investigate force steadiness participants are instructed to follow one-half of a sine curve wavelength by grading the force produced during leg-press. The half wavelength durantion is 20 s and peaks at 35% of MVC based on the average of leg-press MVC trials. The sine curve is displayed on a screen together with real-time display of force trace produced. Participants perform two force steadiness trials which are averaged.

Using a custom build handgrip dynamometer isometric MVC is assessed unilaterally with the participant seated in a chair without armrests, feet flat on the ground, arms along the torso, and holding the dynamometer in a neutral wrist position. The handgrip dynamometer is placed in the hand so that middle phalanx pressed the dynamometer into the palm of the hand. Three trials producing handgrip MVC are performed with each hand or until no increase in maximum force is produced. The trial with the highest MVC is chosen for further analysis.

In the isokinetic dynamometer MVC contractions of the weakest leg, determined from the leg-press test, is assessed. This includes knee extensors, knee flexors, plantar flexors, and dorsal flexors. Assessments are isometric contractions and isokinetic contractions at 60°/s and 180°/s. When assessing knee extension and flexion participants are seated upright with the hip in 85° flexion and the lateral epicondyle of the knee is aligned with the axis of rotation of the lever arm of the dynamometer. A Velcro strap placed 5 cm proximal to the medial malleolus secures the dynamometer arm to the leg of the participant. When assessing plantar flexion and dorsal flexion participants are seated upright with the hip in 50° flexion and the knee in 45° flexion. The bare foot is placed on a footplate, so the lateral malleolus of the ankle is aligned with the axis of rotation of the lever arm of the dynamometer, and then fixed using Velcro straps. For both knee and ankle assessments a shoulder and hip harness are used to fixate the participants, hence minimising extraneous body movement. Three trials are performed or until no increase in maximum force is produced for each condition. The trial with the highest MVC for each condition is chosen for further analysis. From this trial, RFD is also determined at 50 ms and 200 ms relative to the onset of contraction, defined as a torque increase of 2 standard deviations (SD) from baseline torque level at rest.

During the measurements of knee extension in the isokinetic dynamometer, the interpolated twitch technique (ITT) is also applied to determine voluntary activation (VA) of the quadriceps muscle [86,87]. Hereby, given a proxy measure of neural drive activating the quadriceps muscle. The ITT consist of an initial familiarization with electrical stimulation (direct current stimulator, model DS7A, Digitimer Ltd, UK) along with determination of the maximal individual stimulation current. The electric stimulation consists of doublet twitches (200-μs duration, 10-ms interstimulus delay) of the quadriceps muscle using two 5 × 10 cm electrodes (Valutrode Lite, Axelgaard, Denmark) positioned 15 cm above the basis of the patella and 15 cm below the anterior superior iliac crest. The ITT stimulation is applied at the force plateau of the MVC and immediately after the MVC at rest, resting twitch. To calculate VA the following equation is used:

VA = 100 − ((MVC - Force at stim) * (Force at stim / MVC) / Resting twitch force) * 100

Hereby, adjusting for situations where the superimposed stimulation is not delivered exactly at peak MVC [88].

##### 2.6.2.3 DXA

Using dual-energy X-ray absorptiometry (DXA) scan (GE Lunar iDXA, GE Healthcare, Madison, WI), body composition as well as bone mineral density are evaluated. Subjects are DXA scanned on the same time of the day (±1 h) across testing time points. The software package (enCORE software v16.0; GE Healthcare, Madison, WI) is used to determine bone mineral density and t-score, young white female reference population, at whole body, femoral neck, and lumbar spine level; in addition, body weight, fat mass, and fat-free mass is also determined using the same software. Routine quality control of the DXA scanner finds the coefficient of variance to be 0.26%.

##### 2.6.2.4 Blood markers

Blood samples are drawn from an antecubital vein in a resting state. 41,5 ml of whole blood is drawn into three 8.5 mL Vacutainer SST™ II PET tube with clot activator and separating gel (BD, USA), one 8 mL vacutainer K2-EDTA (BD, USA), and two S-monovette K3-EDTA 4 mL (Sarstedt, Germany) with 8 μL protease inhibitor comprised of 1 μg/mL Aprotinin (Roche Diagnostics, Mannheim, Germany), 100 μg/mL mM Pefabloc® SC protector (Roche Diagnostics, Mannheim, Germany) and 0.5 μM KR-62436 hydrate (Sigma-Aldrich, St. Louis, Missouri, United States). After blood is dawn the serum tubes coagulate at room temperature for 30 min and is subsequently centrifuged at 1300 g for 10 min. The serum is then divided into Eppendorf tubes in 1 ml aliquots. EDTA tubes are kept on ice and centrifuged at 1300 g for 10 min at 4 °C immediately after the collection of blood samples. The plasma is then divided into Eppendorf tubes in 1 ml aliquots. Serum and plasma samples is stored at −80 °C until further analyses.

Samples analyses include biomarkers for neurodegenerative factors (neurofilament light chain [89] and glial fibrillary acidic protein [90]), neurotrophic factors (brain-derived neurotrophic factor [91,92] and insulin-like growth factor-1 [92]), immunological factors (interleukin-6, tumor necrosis factor alpha, C-reactive protein [93], and S100 [94]), and bone metabolism (propeptide of type 1 procollagen, beta-C-terminal telopeptide [95], sclerostin [96], 25 OH D-vitamin, calcium, phosphate, and parathyroid hormone [95,97]). Hereby allowing for exploring the effect of PPT on these relevant biomarkers for neurodegeneration, neurotrophy, immune system, and bone health.

##### 2.6.2.5 Cognitive function

The selective reminding test (SRT) measures memory from both long-term storage and consistent long-term storage. The test is conducted and scored according to the original instructions [98]. After 20 min, interspersed by other tests, participants are asked to recall the word list, where the number of words recalled correctly constitutes the delayed recall score. To diminish potential learning effects participants will receive different SRT forms in a randomized order at each testing timepoint.

The symbol digit modalities test (SDMT) is a measure of cognitive processing speed and attention using the translation geo-metric symbol numbers from 1 to 9. The number of correct translations by the participant in 90 s is the score [99]. A change of 4 points or more on the SDMT is considered a clinically relevant change [100]. To get a proxy measure of cognitive fatigability number of correct translations are compared for the first 30 s and last 30 s [101].

##### 2.6.2.6 Physical activity

Physical activity levels in pwMS are lower than in matched HC [102,103]. Physical activity is in the present study objectively measured using an accelerometer (Axivity AX3, Axivity, Newcastle, UK) worn for seven consecutive days. The accelerometer is placed on the mid-anterior thigh of the weaker leg using self-adhesive tape (Fixomull Stretch, BSN Medical, Hamburg, Germany). Data is sampled at 100 Hz with a sensitivity of ±8G. The setup of the accelerometers and the downloading of raw data are done using OmGuiV.1.0.0.43 (Newcastle University, UK). Thigh-worn accelerometers provide more reliable and valid measurements of physical activity compared to other placements. Data analysis is performed using custom-built software (Propero by Jan Christian Brønd, Odense, Denmark) as previously described [104]. Additionally, minutes of sedentary, light intensity, moderate intensity, and vigorous intensity physical activities will be determined based on relevant cut-off levels of counts per minute.

### 2.13 Patient-reported outcomes

The MS impact scale (MSIS) consists of 29 items assessing the impact of MS on the life of a pwMS from the perspective of the patient. The impact of MS is divided into a physical and a psychological component [105,106]. MSIS score ranges from 29 to 145 points (high scores indicating a larger impact of MS), and a change of 7 points is considered clinically relevant [107].

The MS walking scale (MSWS) assesses the impact of MS on walking abilities and mobility in pwMS via a 12-items questionnaire, where each item is scored from 1 to 5 [108]. The MSWS score is transformed to a percentage score (0–100) of the maximum possible score (a higher score indicating larger impact of MS on walking abilities), and a change of 10.7 points is considered clinically relevant [74].

The modified fatigue impact scale (MFIS) assesses the subjective perception of fatigue impact, which is among the most frequent and disabling symptoms of MS [109]. The MFIS questionnaire score ranges from 0 to 84, with higher scores indicating greater fatigue impact. A score of ≥38 points indicates clinical fatigue [110], and a change of 4 points is considered clinically relevant [111]. The Danish version of MFIS has been validated and shows good reliability [112].

The hospital anxiety and depression scale (HADS) measures emotional distress via 14 items with seven items probing anxiety and seven items probing depression. Each item is scored from 0 to 3 with higher scores representing more emotional distress [113]. HADS was developed for use in medically ill patients and has later been validated in pwMS [114].

The euro quality of life 5-dimension (EQ-5D) is a non-disease-specific questionnaire used to determine quality of life [115,116]. The EQ-5D has previously been used in MS populations [117].

The falls efficacy scale-international (FES-I) is a questionnaire assessing concern of falling when performing different activities and is validated in pwMS [118]. It consists of 16 items which are rated from 1 to 4, where a higher score indicates a greater concern of failing [119]. Additionally, a 12-months falls recall (number of falls, injurious falls) are carried out [120].

The Pittsburgh sleep quality index (PSQI) assesses the subjective sleep quality in a 19-item questionnaire. The total score (global PSQI score) is the sum of all components (range: 0–21): a score ≥5 represents poor sleepers; <5 represents participants with normal sleep quality [121,122].

The Brief Pain Inventory (BPI) measures the level of self-reported pain. The short form of the BPI is used, which consists of 9 items concerning different aspects of pain rated from 0 to 10 [123,124].

### 2.7 Data handling

All digital data, except questionnaires, will be visually inspected for quality at the time of collection and stored in accordance with the rules defined by the Danish Data Protection Agency. All analogue data will undergo double data entry and will be stored in accordance with the rules defined by the Danish Data Protection Agency both in analogue and digital form. All data will be entered pseudonymized and the decryption key will be stored separately from the data. Access to data is provided by Aarhus University.

### 2.8 Statistics

Characterization of the study sample across groups will be carried out using multiple baseline demographic and anthropometric variables (reported as number of observations, mean ± SD or median ± IQR, and proportions were appropriate). Data is checked for normality via visual inspection of histograms and quantile-quantile plots. In case of non-normality, appropriate data transformations will be performed. To investigate the effect of the 24 weeks PPT intervention and the 24 weeks follow-up period on the primary outcome (i.e., whole brain atrophy rate), a linear mixed model with repeated measures is used with group and time as fixed effects and participant id as random effects. The secondary outcomes will be analyzed analogous to the primary outcome. The analyses are performed following the intention-to-treat principle (i.e., all participant data are included, but without use of “last observation carried forward”) as the mixed model allows for missing value. In addition, a per-protocol analysis is performed based on adherence levels (only including participants that attend ≥80% of the planned PPT training sessions). An alpha level of 0.05 is used for all comparisons.

### 2.9 Ethics and dissemination

The study will be performed in accordance with the principles of the Declaration of Helsinki. Further, the study is approved by the Central Denmark Region Committees on Health Research Ethic with reference number 1-10-72-222-20 and the Danish Data Protection Agency with reference number 2016-051-000001. Reporting of results will follow the Consolidated Standards of Reporting Trials statement [125] and will be published by the investigators in relevant scientific peer-reviewed journals, independent of study findings. Authorship eligibility will follow the Vancouver recommendations. Besides publication in scientific peer-reviewed journals, the study results will be presented at scientific conferences and communicated to patients through the Danish MS Society. Adverse events will be registered and reported to the Central Denmark Region Committees on Health Research Ethics according to the existing guidelines.

## 3 Discussion and perspectives

We have presented the protocol for a trial that evaluates the neuroprotective effect of PPT in older pwMS, specifically evaluating whole brain atrophy rate (primary outcome). To our knowledge, neither PPT nor exercise in general have previously been investigated for its effect on brain morphology in older pwMS, despite this population constituting approximately one-third of all pwMS [[7], [8], [9]]. The presented study secondarily evaluates the effects of PPT on macro and micro brain structures, neuromuscular function, physical function, cognitive function, bone health and patient-reported outcomes, which we believe will expand the current knowledge of PPT in pwMS [36].

A limitation of the presented study is that the primary outcome (i.e., whole brain atrophy rate) has recently been shown to remain almost unaffected following 24–48 weeks of aerobic exercise in two large RCT's. In contrast, PRE, although sparsely investigated, have shown positive effects on CNS morphology in one pilot RCT by Kjølhede et al. [41]. Speculatively, the difference in exercise modality and hence physiological response could account for these discrepancies in the effect of exercise on CNS morphology.

Another potential limitation regarding the primary outcome is that the power calculation is based on an expected dropout rate of 16%. The lack of studies investigating PPT in older pwMS limits our knowledge of dropout in this subpopulation of pwMS. In young and middle-aged pwMS a dropout of 0–47 % has been reported across exercise interventions and adherence to progressive resistance exercise interventions of 91 % is found in a systematic review and meta-analysis [126]. Therefore, the presented study could be at risk of having reduced statistical power to detect true changes if the dropout exceeds the 16 % applied in the power calculation. Further, if changes are detected there is limited knowledge on how large a change in global brain atrophy rate is needed to make a clinically meaningful change. However, it is assumed that any reduction in global brain atrophy rate is valuable.

The presented study is expected to be finalized mid-2024, and the findings have the potential to guide clinically meaningful interventions for older pwMS.

## Funding statement

The study is financed by Aarhus University, Faculty of Health (Vennelyst Boulevard 4, 8000 Aarhus C, DK, health@au.dk) and by the following external foundations: 'Trygfonden', The Danish MS Society 'Scleroseforeningen', ’Jascha fonden’, ’Helsefonden’, and The Danish Osteoporosis Society ’Osteoporose foreningen’.

None of the funders has a role in the design, management, analysis, interpretation of data, writing, or publication of results.

## CRediT authorship contribution statement

**Tobias Gaemelke:** Conceptualization, Funding acquisition, Methodology, Visualization, Writing – original draft, Writing – review & editing. **Christoffer Laustsen:** Conceptualization, Methodology, Writing – review & editing. **Peter Feys:** Conceptualization, Writing – review & editing. **Lars Folkestad:** Conceptualization, Methodology, Writing – review & editing. **Marianne Skovsager Andersen:** Conceptualization, Methodology, Writing – review & editing. **Niklas Rye Jørgensen:** Conceptualization, Methodology, Writing – review & editing. **Marie-Louise Jørgensen:** Conceptualization, Funding acquisition, Methodology, Writing – review & editing. **Sune Nørhøj Jespersen:** Conceptualization, Methodology, Writing – review & editing. **Steffen Ringgaard:** Conceptualization, Methodology, Writing – review & editing. **Simon F. Eskildsen:** Conceptualization, Methodology, Writing – review & editing. **Ulrik Dalgas:** Conceptualization, Funding acquisition, Methodology, Writing – review & editing. **Lars G. Hvid:** Conceptualization, Funding acquisition, Methodology, Writing – review & editing.

## Declaration of competing interest

The authors declare that they have no known competing financial interests or personal relationships that could have appeared to influence the work reported in this paper.
